# Progress on 3-Nitropropionic Acid Derivatives

**DOI:** 10.3390/biom15081066

**Published:** 2025-07-24

**Authors:** Meng-Lin Feng, Zheng-Hui Li, Bao-Bao Shi

**Affiliations:** 1 School of Pharmaceutical Sciences, South-Central Minzu University, Wuhan 430074, China; 2023110476@mail.scuec.edu.cn (M.-L.F.); 2015051@mail.scuec.edu.cn (Z.-H.L.); 2 International Cooperation Base for Active Substances in Traditional Chinese Medicine in Hubei Province, School of Pharmaceutical Sciences, South-Central Minzu University, Wuhan 430074, China

**Keywords:** 3-Nitropropionic acid derivatives, pharmacological activity, natural products, neurotoxicity, antiviral activity

## Abstract

3-Nitropropionic acid (3-NPA) is a deadly neurotoxic nitroalkane found in numerous fungi and leguminous plants. 3-NPA, known as an antimetabolite of succinate, irreversibly inhibits succinate dehydrogenase and disrupts mitochondrial oxidative phosphorylation. Its utility in modeling Huntington’s disease (HD) and oxidative stress has garnered significant research interest. Derivatives of 3-NPA, formed through esterification, have a wide range of biological activities including neurotoxic, antiviral, insecticidal, antimicrobial and antioxidant properties. This review systematically summarizes the structural characteristics, biological activities, and chemical synthesis of 3-NPA-derived compounds, providing valuable insights for further research and therapeutic applications.

## 1. Introduction

**Figure 1:**
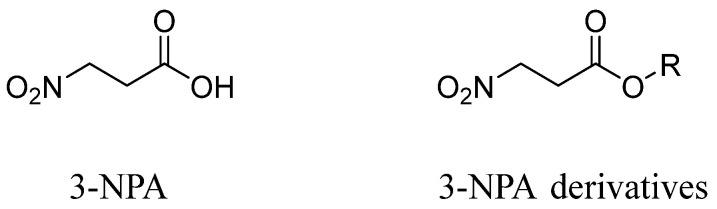
Chemical structures of 3-Nitropropionic acid and its derivatives.

3-Nitropropionic acid (3-NPA) (**1**) (Figure 1) is a potent neurotoxic nitroalkane initially isolated from fungi such as *Aspergillus flavus*, *Aspergillus oryzae*, and *Arthrinium* spp. and is occasionally found in some leguminous plants [1,2,3]. Compound **1**, known as an antimetabolite of succinate, irreversibly inhibits succinate dehydrogenase and disrupts mitochondrial oxidative phosphorylation. While **1** was first reported in 1920 [4], its structure was not elucidated until 1949 [5]. In 1959, Britten, E. J. identified 3-NPA as the major toxic compound of indigo by comparing sodium nitrite and 3-NPA [6]. In China, nearly 1000 cases of acute encephalopathy and gastrointestinal disorders (e.g., nausea, vomiting, and abdominal pain) were linked to moldy sugarcane consumption, with severe cases resulting in death or delayed dystonia [7]. Researchers isolated *Arthrinium* spp. from moldy sugarcane and identified 3-NPA as the causative toxin [8]. In 1991, Ludolph et al. [9] investigated the pathological mechanisms underlying 3-NPA-mediated human mortality and delayed-onset dystonia. Their research revealed 3-NPA’s neurotoxic properties in experimental animals, leading to the proposal of a standardized animal model for studying selective neuronal metabolic damage. Subsequently, animal models based on 3-NPA toxicity emerged as a prominent research focus. 3-NPA has been primarily utilized to study therapeutic interventions across various symptomatic stages of Huntington’s disease (HD). Furthermore, pathological investigations of HD and 3-NPA have established animal models characterized by mitochondrial morphological alterations, cerebral hypoxia, caspase-3 activation, bradykinesia, and memory impairment [10,11,12,13,14]. 3-NPA HD models could be a better option for screening novel drugs to manage HD [15]. The derivatives of 3-Nitropropionic acid exhibit toxicity similar to 3-NPA. Carter et al. [16] first reported the lethality of karaka fruit to livestock and isolated 3-NPA and its glycoside derivative karakin (**3**), confirming its role as a primary toxin. Because the prolonged consumption of the seeds of some species of the genus *Lathyrus* (Leguminosae), e.g., *L. sativus*, can cause lathyrism, a neuronal disease, many of the secondary metabolites in these plants, such as those derived from isoxazolin-5-one and 3-NPA, have been characterized in terms of their neurotoxicity [17]. Subsequently, toxicological investigations of 3-Nitropropionic acid derivatives emerged as a prominent research focus. Byers et al. [18] further demonstrated that the majority of these derivatives exhibit potent insecticidal and antifeedant activities, along with neuroactive effects beyond classical toxicological mechanisms. The neuroregulatory properties of these compounds were elucidated by Acheson’s group [19]. Notably, *α*-L-galactopyranose 2,3,4,6-tetrakis (3-nitropropanoate) was found to specifically inhibit the binding of the neurotrophic precursor protein p75 receptor and disrupt the cellular internalization of pro-nerve growth factor (proNGF).

**Scheme 1:**
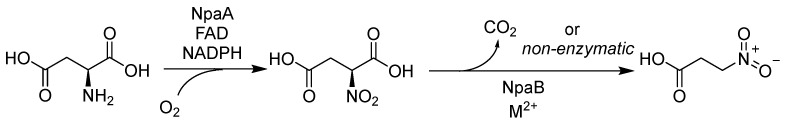
Biosynthetic pathways of 3-Nitropropionic acid.

In 2024, Tang, Y’s group elucidated the biosynthetic pathway of 3-nitropropanoic acid (3-NPA) in the koji mold *Aspergillus oryzae* [20] (Scheme 1). The identified biosynthetic gene cluster (BGC) contains genes encoding an amine oxidase, NpaA, and a decarboxylase, NpaB. Genetic knockout experiments confirmed that NpaA functions as an (S)-nitrosuccinate synthase, catalyzing the oxidation of L-aspartate to (S)-nitrosuccinic acid. The decarboxylase NpaB potentially accelerates the subsequent decarboxylation step. Notably, a conserved BGC for 3-nitropropanoic acid was identified across various fungi used in food processing, despite the absence of reported 3-NPA production in these organisms [20].

Most 3-Nitropropionic acid derivatives are small-molecule compounds with significant potential in the development of novel therapeutics and biomedical applications. This review categorizes known derivatives based on three aspects: structural features, physiological activities, and biosynthetic pathways, aiming to facilitate future studies.

## 2. Structural Classification of 3-Nitropropionic Acid Derivatives

3-NPA derivatives are compounds formed through the dehydrogenation of the carboxylic acid group in 3-NPA. Based on the esterified groups, these compounds are classified into three categories: 3-NPA sugar esters, 3-NPA non-sugar esters, and other 3-NPA derivatives.

## 3. 3-Nitropropionic Acid Sugar Esters

3-NPA sugar esters are a class of compounds synthesized through the dehydration condensation of sugar (or a derivative of sugar) with 3-NPA via esterification. This classification represents the largest group of 3-NPA derivatives with 44 compounds, accounting for approximately 80% of all reported compounds. These 3-Nitropropionic acid sugar esters are predominantly isolated from higher plants, with a minor fraction derived from fungi and insects.

**Figure 2:**
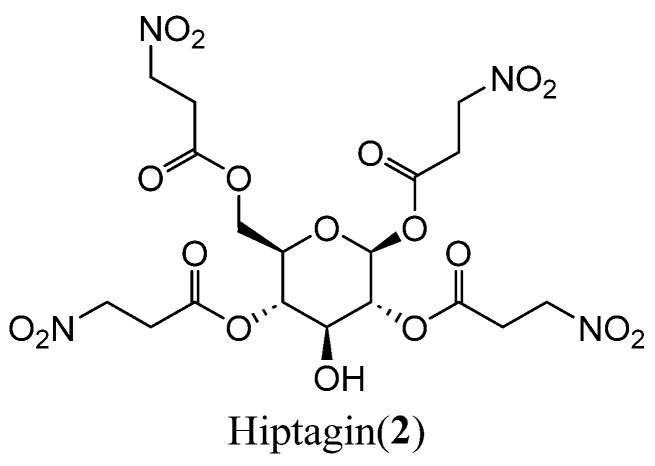
Structure of compound **2**.

In 1920, Gorter [4] first isolated a 3-Nitropropionic acid glycoside, Hiptagin (**2**) (Figure 2), from *Hiptage benghalensis* Kurz. Gnanasunderam et al. [21] also isolated compound **2** from *Lotus pedunculatus* (Fabaceae), a Fabaceae species native to Europe. Utilizing feeding bioassays, they demonstrated that compound **2**, the bioactive compound derived from this plant, exhibits potent antifeedant activity against the third-instar larvae of *Costelytra zealandica*, a key subterranean pest in New Zealand. This discovery facilitated the development of botanical insecticides targeting *C. zealandica* and related soil-dwelling pests. Mechanistic studies of Hiptagin’s activity have provided critical insights for designing eco-friendly pest control strategies. A research group at the University of Sheffield [22] elucidated 75 compounds that exhibit therapeutic potential for neurodegenerative disorders. Notably, compound **2** was characterized as a potent activator of the Nrf2-ARE pathway, demonstrating applicability in managing oxidative stress-mediated pathologies, including motor neuron diseases.

**Figure 3:**
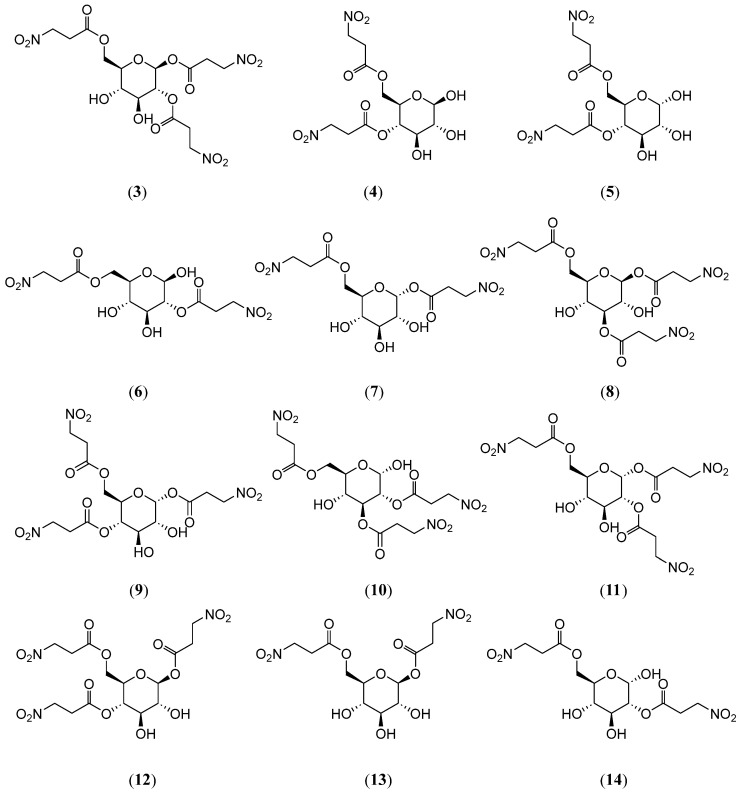
Structures of compounds **3**–**14**.

Pfeffer et al. [23] characterized seven 3-Nitropropionic acid sugar esters, including 4,6-di-*O*-(3-nitropropanoyl)-*β*-D-glucopyranose (**4**), 4,6-di-*O*-(3-nitropropanoyl)-*α*-D-glucopyranose (**5**), 2,6-di-*O*-(3-nitropropanoyl)-*β*-D-glucopyranose (**6**), 1,6-di-*O*-(3-nitropropanoyl)-*α*-D-glucopyranose (**7**), 1,3,6-tri-*O*-(3-nitropropanoyl)-*β*-D-glucopyranose (**8**), and 1,4,6-tri-*O*-(3-nitropropanoyl)-*α*-D-glucopyranose (**9**) (Figure 3). Furthermore, Moyer et al. [24] isolated five additional 3-Nitropropionic acid sugar esters, including karakin (**3**), corollin (**10**), coronillin (**11**), corynocarpin (**12**), cibarian (**13**), and coronarian (**14**) (Figure 3), from *Coronilla varia* L. (Fabaceae). Through ^13^C-NMR spectroscopy and linear regression analysis of ^13^C substitution parameters at varying esterification sites on the *α*/*β*-glucopyranose scaffold, two distinct sets of chemical shift constants were derived. The theoretical chemical shifts calculated using these parameters exhibited high congruence with experimental data. Furthermore, the inductive and electron affinity effects of the substituents were correlated with their esterification positions and spatial orientations. These parameters were subsequently utilized to elucidate the structures of additional derivatives.

Byers et al. [18] also isolated 3-NPA, karakin (**3**) (Figure 3), compound **11**, and compound **13** from *Coronilla varia* L. (Fabaceae). They employed *Trichoplusia ni*, a polyphagous species with an extensive host range, as the experimental model organism, observing that 3-NPA and its glycoside derivatives significantly increased mortality in first-instar larvae, with toxicity ranked in the following order: 3-NPA > trisubstituted derivatives > disubstituted derivatives. These compounds also reduced pupal weight and impaired reproductive capacity in *Trichoplusia ni* although egg hatchability remained unaffected. Byers et al. [25] further demonstrated that 3-NPA, compound **3**, and compound **13** exhibited toxicity not only in non-ruminants (e.g., chickens and pigs) but also significantly increased mortality and reduced pupal weight in Spa-rganothis fruitworm larvae. Kigel [26,27] reported that the intravenous administration of compound **11** in cats at 0.03 mg/kg induced negative chronotropic effects (heart rate reduction) and positive inotropic effects (myocardial contractility enhancement), with a duration of action lasting 6 h, comparable to cardiac glycosides like Gophruzid and Periplocin. These findings confirm that compound **11** shares the hallmark cardiotonic properties of cardiac glycosides.

**Figure 4:**
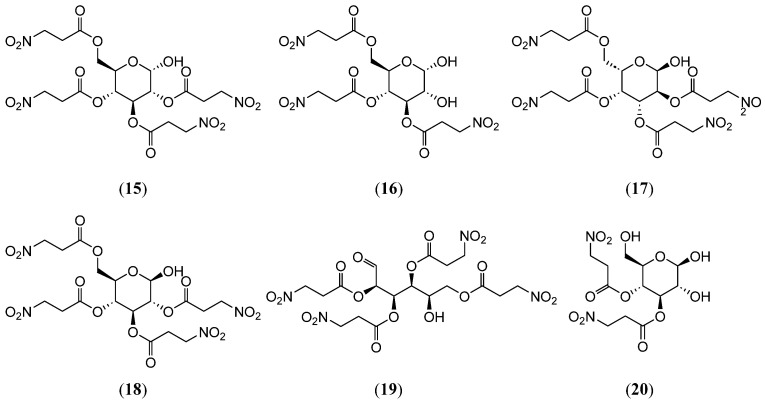
Structures of compounds **15**–**20**.

Benn et al. [28] isolated three 3-Nitropropionic acid sugar esters, karakin (**3**), 2,3,4,6-tetra-*O*-(3-nitropropanoyl)-*α*-D-glucopyranose (**15**), and 3,4,6-tri-*O*-(3-nitropropanoyl)-*α*-D-glucopyranose (**16**) (Figure 4), from *Indigofera linnaei* (Fabaceae). A research group at Cornell University [29] identified that the 2,3,4,6-tetra-*O*-(3-nitropropanoyl)-*α*-L-galactopyranose (**17**) (Figure 4) inhibits proNGF signaling by suppressing its cellular internalization. The experimental data revealed a 70.96% reduction in proNGF uptake. This compound’s ability to disrupt the cellular trafficking of neurotrophic precursor proteins positions it as a candidate for treating neurological diseases characterized by apoptotic pathways and aberrant neurotrophic factor signaling.

Roman et al. [30] isolated 2,3,4,6-tetra-*O*-(3-nitropropanoyl)-*β*-D-glucopyranose (**18**) (Figure 4), a 3-nitropropanoate acid derivative, from the root extracts of the endemic Brazilian species *Heteropteris aphrodisiaca*. Antibacterial susceptibility testing revealed the broad-spectrum activity of this compound against *Staphylococcus aureus*, *Bacillus subtilis*, *C. albicans*, *C. tropicalis*, *C. parapsilosis*, and *C. krusei.* Moreover, researchers at the Federal University of São Paulo [31] identified D-Glucose, 2,3,4,6-tetrakis (3-nitropropanoate) (**19**) (Figure 4), a structurally analogous 3-Nitropropionic acid glycoside, from the same botanical source. This compound displayed a minimum inhibitory concentration (MIC) of 250 μg/mL and a minimum bactericidal concentration (MBC) of 500 μg/mL against *S. aureus*, alongside an MIC/MBC profile of 125/250 μg/mL for *B. subtilis*. Furthermore, this compound exhibited marked antifungal efficacy against *C*. *albicans*, *C. tropicalis*, and *C. parapsilosis* and demonstrated dose-dependent antiviral activity against the herpes simplex virus (HSV) and poliovirus, achieving inhibition rates of 69–94% within a concentration range of 12.5–50 μg/mL.

**Figure 5:**
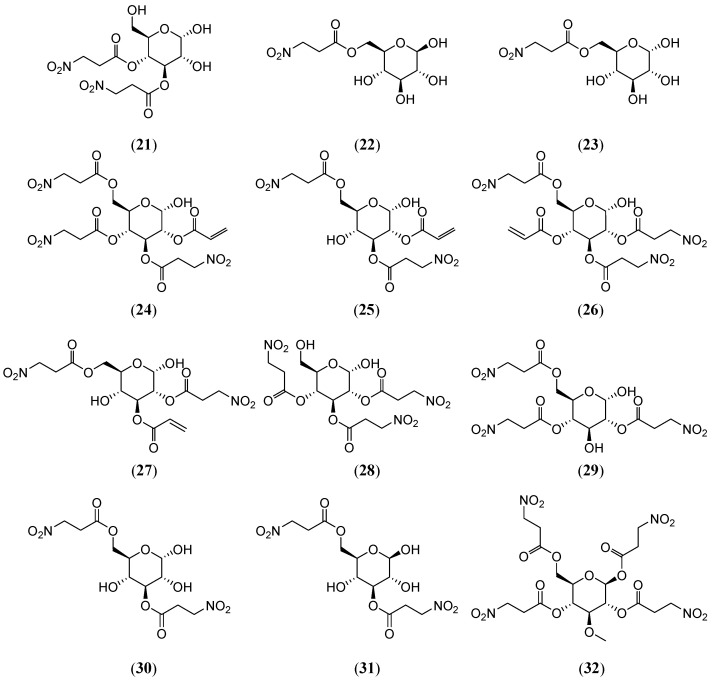
Structures of compounds **21**–**32**.

*Indigofera kirilowii*, a plant native to the Shaanxi, Henan, Hubei, and Shanxi provinces, is primarily used as a source of the traditional Chinese medicine Shandougen (*Euchresta japonica* Benth. ex Oliv.) and is known for its heat-clearing and detoxifying properties. However, the substitution of Shandougen with *I. kirilowii* roots has led to neurotoxicity in some patients, with severe cases resulting in death. Thus, investigating the chemical composition of *I. kirilowii* root is critical for identifying its toxic components. Yang, et al. [32] isolated four 3-Nitropropionic acid sugar esters from the roots of *I. kirilowii* (Fabaceae): 3,4-di-*O*-(3-nitropropanoyl)-*β*-D-glucopyranose (**20**) (Figure 4), 3,4-di-*O*-(3-nitropropanoyl)-α-D-glucopyranose (**21**), 6-*O*-(3-nitropropanoyl)-*β*-D-glucopyranose (**22**), and 6-O-(3-nitropropanoyl)-*α*-D-glucopyranose (**23**) (Figure 5). Subsequent studies [33,34] isolated 10 additional 3-Nitropropionic acid sugar esters (kirilowin A-H (**24–31**)) (Figure 5) from the same plant. Notably, *β*-D-Glucopyranose (**22**), originally isolated from *Securigera varia* (Fabaceae) by Sientzoff et al. [35], demonstrated DPPH radical scavenging activity with an EC_50_ value of 133.5 ± 2.8 μg/mL, indicating significant antioxidant potential. Finnegan et al. [36] synthesized the tetra-acylated derivative Hiptagin methyl ether (**32**) (Figure 5) via the acylation of 3-O-methyl-*α*-D-glucopyranose with 3-nitropropanoyl chloride in N-methylpyrrolidone solution.

**Figure 6:**
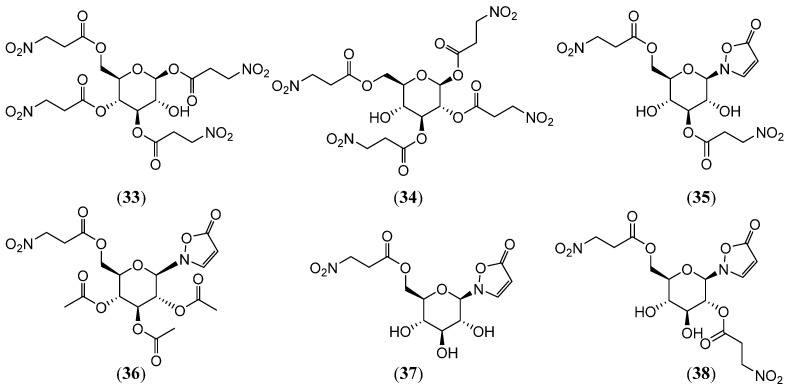
Structures of compounds **33**–**38**.

Majak et al. [37] isolated seven compounds from the New Zealand plant *Corynocarpus laevigatus*: compounds **2**, **8**, **16**, and **22–23**, 1,3,4,6-Tetrakis-*O*-(3-nitropropanoyl)-*β*-D-glucopyranose (**33**), and 1,2,3,6-Tetrakis-*O*-(3-nitropropanoyl)-*β*-D-glucopyranose (**34**) (Figure 6). Sugeno et al. [38] identified five 3-Nitropropionic acid derivatives in the secretions of four Japanese *Chrysomelidae* beetles: compounds **3** and **13**, 2-[3′,6′-di-(3″-nitropropanoyl)-*β*-D-glucopyranosyl]-3-isoxazolin-5-one (**35**), 2-[2′,6′-di-(3″-nitropropanoyl)-*β*-D-glucopyranosyl]-3-isoxazolin-5-one (**36**), and 2-[6′-(3″-nitropropanoyl)-*β*-D-glucopyranosyl]-3-isoxazolin-5-one (**37**) (Figure 6). Among these, compounds **3**, **13**, **35**, and **36** exhibited potent repellent effects against *Tetramorium caespitum* (*p* < 0.01). Compounds **3** and **36** showed significant repellency at the tested concentrations (*p* < 0.05), highlighting their roles in the defense mechanisms of *Chrysomelidae* beetles and their potential as natural repellents. Pasteels et al. [39,40] isolated compounds **37** and 2-[2,3,4-tri-*O*-acetyl-6-*O*-(3-nitro-1-oxopropyl)-*β*-D-glucopyranosyl]-5(2H)-isoxazolone (**38**) (Figure 6) from the eggs, neonate larvae, and defensive secretions of beetles within the *Chrysomelina* and *Phyllo-dectina* subfamilies. Bioassays demonstrated that compound **37** had a significant repellent effect on red ants (*Myrmica rubra*) at the concentration found in the eggs (about 10^−2^ M); this repellent effect persisted at lower concentrations (10^−3^ M) and higher concentrations. The above results suggest that, in *Chrysomelina* subfamilies, isoxazolone (**37**) protects eggs from predators through its strong repellent effects. Concurrently, compound **38**, identified in adult secretions, functions as a defensive compound, mediating predator avoidance and thereby facilitating ecological fitness in chrysomelid beetles.

**Figure 7:**
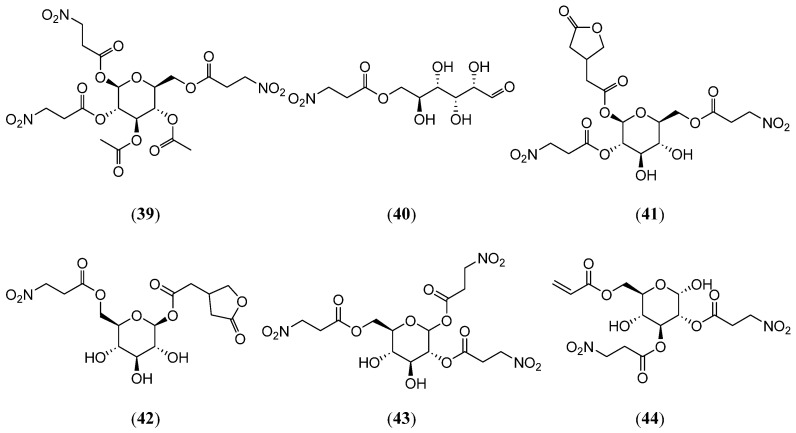
Structures of compounds **39–44**.

Carter et al. [41] developed a protocol for synthesizing and purifying compound **3** and its derivative, compound **12**, involving crystallization, acetylation, hydrolysis, and chromatographic separation. Notably, the structural elucidation of compound **12** was further confirmed by synthesizing its acetylated derivative, diacetylkarakin (**39**) (Figure 7).

*Astragalus canadensis*, the most widespread *Astragalus* species in North America, exhibits toxicity to sheep and cattle [42]. Benn et al. [43] identified seven 3-Nitropropionic acid sugar esters in this plant: compounds **3**, **8**, **12**, and **13** and D-Glucose, 6-(3-nitropropanoate) (**40**), 1-*O*-[5-oxotetrahydrofuran-3-yl]acetyl-2,6-di-*O*-(3-nitropropanoyl)-*β*-D-glucopyranose (**41**), and 1-*O*-[5-oxotetrahydrofuran-3-yl]acetyl-6-*O*-(3-nitropropanoyl)-*β*-D-glucopyranose (**42**) (Figure 7). The hydrolysis of these glycosides releases 3-NPA, amplifying the plant’s toxicity.

Three 3-Nitropropionic acid sugar esters, compounds **13–14**, and 1,2,6-tri-(3-nitropropanoyl)-*α*-D-glucopyranose (**43**) were isolated from *Coronilla varia* (Fabaceae) by Gustine et al. (Figure 7) [44]. In vitro and in vivo experiments confirmed that rumen microbiota metabolize these glycosides and that compound **43** was rapidly hydrolyzed to 3-NPA and glucose within 24 h, with 3-NPA further degrading to unknown metabolites. When sheep ate *C. varia*, the 3-NPA in their rumen completely disappeared within 6 h without any signs of toxicity. Voles did not show symptoms of toxicity after consuming the rumen-fluid-treated *C. varia*, indicating that the 3-Nitropropionic acid sugar esters were successfully detoxified via rumen microorganisms. Zhang et al. [45] isolated and characterized two novel aliphatic nitro compounds, compound **25** and 6-*O*-acryl-2,3-di-(3 nitropropanoyl)-*α*-D-glucopyranose (**44**) (Figure 7), from *Indigofera carlesii* (Fabaceae), providing new chemical insights for exploring its medicinal potential.

## 4. 3-Nitropropionic Acid Non-Sugar Esters

3-Nitropropionic acid non-sugar esters are organic compounds formed through the esterification of 3-NPA carboxylic acid, linked via single, double, or triple bonds to form linear or branched structures. Linear derivatives are primarily used in chemical synthesis. Despite their simple structures, they exhibit diverse functionalities and are applied in materials science, pharmaceuticals, and agriculture. Investigating their structural and physicochemical properties can facilitate the development of compounds with enhanced practical applications.

**Scheme 2:**
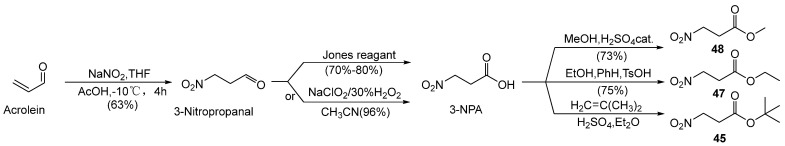
Preparation of compounds **45**, **47**, and **48**.

Zhang et al. [46] synthesized *tert*-butyl 3-nitropropionate (**45**) (Scheme 2) via a three-step process, starting with the nitration of acrolein to yield 3-nitropropanal, followed by the oxidation of 3-nitropropanal to 3-NPA and finally the esterification of 3-NPA with isobutylene catalyzed via concentrated sulphuric acid. The overall isolated yield was 30%.

**Scheme 3:**
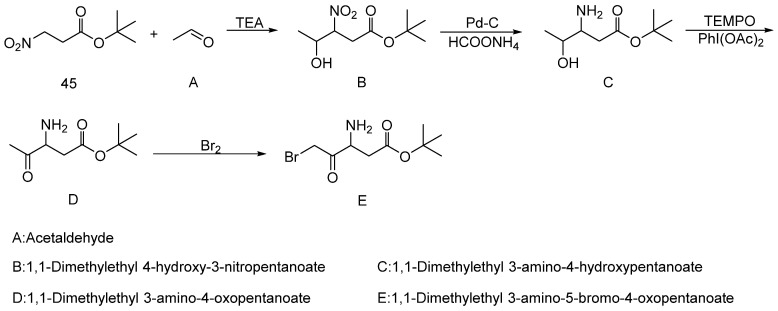
Preparation of 1,1-dimethylethyl 3-amino-5-bromo-4-oxopentanoate.

A research group from the China Pharmaceutical University [47] identified *tert*-butyl 3-nitropropionate (**45**) (Scheme 3) as a key starting material for synthesizing the emricasan intermediate 1,1-dimethylethyl 3-amino-5-bromo-4-oxopentanoate, an inhibitor of the Caspase family of cysteine proteases. The nitro and *tert*-butoxy ester groups in its structure provide critical functional groups for subsequent reactions. This synthetic route offers advantages such as readily available raw materials, mild reaction conditions, and high yields, making it suitable for industrial-scale production. The overall yield was 52.1%.

**Scheme 4:**
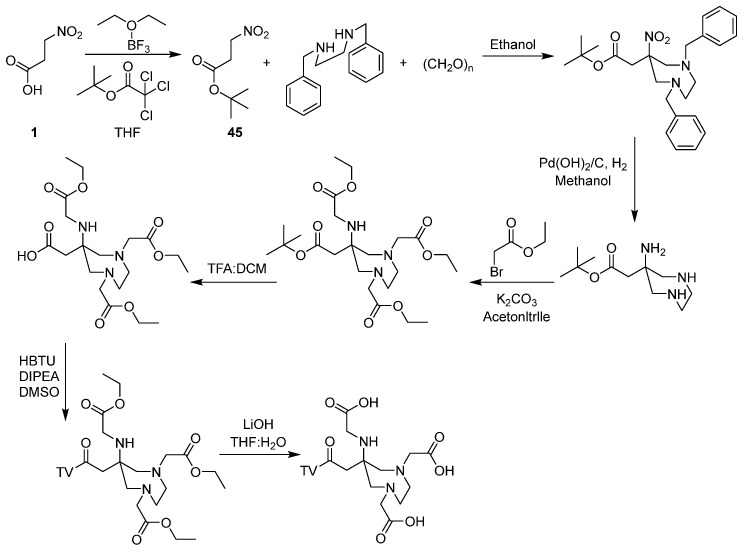
Preparation of BFCs from compound **45**.

Compound **45** serves as a key synthetic intermediate for introducing nitro and carboxyl groups, constructing molecular scaffolds, and serving as a protecting group in the synthesis of bioactive compounds. Through sequential reactions, it is converted into ICE/CED-3 family inhibitors that suppress apoptosis. These inhibitors have significant applications in expanding hematopoietic cell populations, prolonging organ transplant survival and enhancing bioproduction efficiency [48,49,50,51,52,53]. Roesch et al. [54] demonstrated that compound **45** acts as a key intermediate in synthesizing bifunctional chelating agents (BFCs) to construct 1,4-diazepane (DAZA) scaffolds. These BFCs are used to chelate radiometals, primarily in non-invasive molecular imaging applications (Scheme 4).

**Scheme 5:**
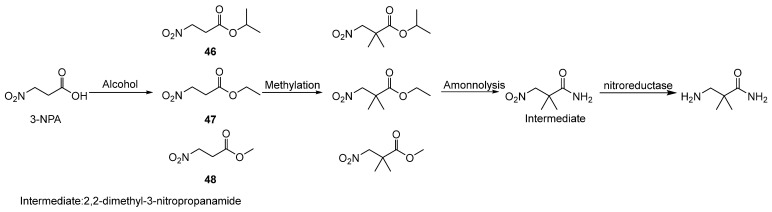
Preparation of key intermediate 2,2-dimethyl-3-aminopropionamide.

Xu [55] presented an enzymatic method for synthesizing the key intermediate of aliskiren (2,2-dimethyl-3-aminopropionamide). The esterification reaction employs isopropyl 3-nitropropanoate (**46**), ethyl 3-nitropropionate (**47**), or methyl 3-nitropropionate (**48**) (Scheme 5) as the critical intermediates, synthesized via the esterification of 3-nitropropanoic acid with aliphatic alcohols (e.g., isopropanol, ethanol, or methanol, with methanol being the preferred substrate). These intermediates serve as essential precursors for subsequent methylation reactions. This enzymatic approach reduces reliance on the high-temperature and high-pressure conditions in traditional synthesis, minimizes equipment and operational demands, and ensures the high purity and yield of the antihypertensive drug intermediate. The yield of the final enzymatic synthesis step is 92%.

**Scheme 6:**
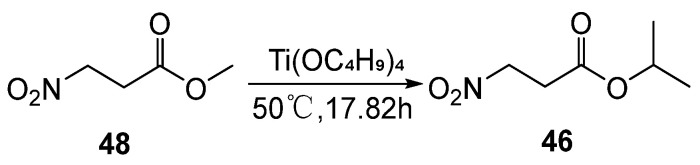
Titanate-catalyzed transesterification reaction.

Seebach et al. [56] introduced a titanate-catalyzed transesterification method for synthesizing diverse functionalized esters. For example, compound **48** can be converted to compound **46**. This method features mild reaction conditions, high-functional group tolerance, strong selectivity, and high product purity, highlighting its potential utility in organic synthesis (Scheme 6). The yield was 50%.

Silva et al. [57] developed a simple, rapid, and high-yield synthetic route to produce 3-NPA and compounds **47** and **48** from commercially available acrolein in two steps, addressing the demand for large-scale preparation (Scheme 2).

Su et al. [58] isolated compound **47** and five 3-Nitropropionic acid sugar esters from the roots of *Indigofera kirilowii* (Fabaceae). Finnegan et al. [59] isolated toxic compound 3-NPA and compounds **2** and **47** from *Indigofera endecaphylla* (Fabaceae). Herdwiani et al. [60] isolated compound **47** from *Cinnamomum burmanni* (Nees and T. Nees) Blume (Lauraceae). Sarkar et al. screened 10 fungal metabolites including compound **47** from *Aspergillus flavus* and *Aspergillus oryzae* as inhibitors of the SARS-CoV-2 main protease. Molecular docking simulations revealed that compound **47** exhibited a docking score of −5.1 kcal/mol with main protease 6LU7, which is comparable to chloroquine (−6.29 kcal/mol), suggesting its potential as an effective antiviral agent. Ma et al. [61] demonstrated that the addition of an isocitrate lyase-specific inhibitor effectively reduced succinic acid accumulation while improving lactic acid purity and productivity. Dose–response experiments revealed that ethyl 3-nitropropanoate (0–0.1 mM) at 0.1 mM decreased succinic acid levels from 2000 ppm to 215 ppm, corresponding to an 89.25% inhibition efficiency. These findings highlight ethyl 3-nitropropanoate’s capacity to selectively suppress succinic acid biosynthesis at sub-millimolar concentrations without compromising lactic acid fermentation kinetics.

Somanthan et al. [62] isolated 3-NPA and compound **48** from *Astragalus* spp. (Fabaceae). Swargiary et al. [63] isolated compound **48** from *Hydrocotyle sibthorpioides* Lam. (Araliaceae) using GC-MS. SwissADME and ADMETlab predictions indicated compound **48**’s favorable pharmacokinetics, low toxicity, and drug-likeness. Its binding affinity to *α*-amylase and *α*-glucosidase suggests potential hypoglycemic activity through enzyme inhibition. Safdar, Sahar et al. identified compound **48** in the ethanol extract of *Selenicereus undatus* (Cactaceae). Antibacterial assays confirmed its antibacterial activity, suggesting its role in the plant’s observed antibacterial and anticancer properties.

**Scheme 7:**
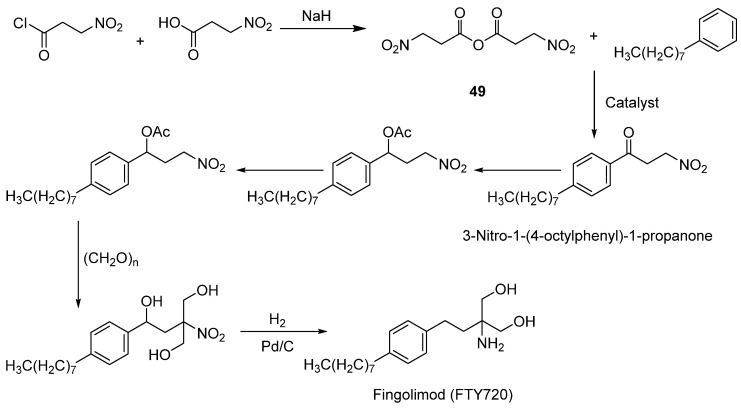
Preparation of fingolimod.

Marom et al. [64] employed 3-Nitropropionic anhydride (**49**) (Scheme 7) as an acylating agent to synthesize fingolimod (FTY720) and its pharmaceutical salts. Compared with traditional acylation methods (e.g., using carboxylic acids and dehydrating agents), this approach achieved higher reaction efficiency and selectivity. Compound **49**, serving as a pivotal starting reagent, facilitates a streamlined synthesis of FTY720 via its high-yielding and regioselective acylation protocols. This methodologically advanced approach enhances reaction kinetics, reduces procedural complexity, and elevates product purity, underscoring its substantial industrial utility in large-scale pharmaceutical manufacturing.

**Scheme 8:**
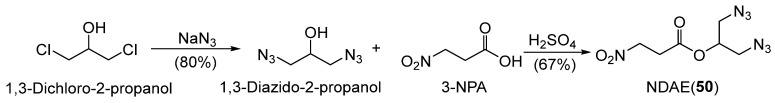
Preparation of compound **50**.

Jin et al. [65] synthesized a novel aliphatic energetic plasticizer, NDAE (**50**) (Scheme 8), by introducing nitro, azido, and ester groups into an aliphatic framework. This compound exhibits low viscosity, low glass-transition temperature, reduced sensitivity, and excellent safety properties, making it suitable for applications in explosives and propellant formulations.

**Figure 8:**
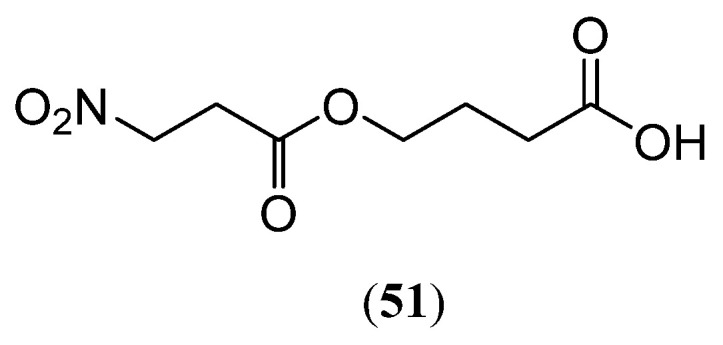
Structure of compound **51**.

Salem et al. [66] surveyed 148 angiosperm species (primarily European) and found a limited distribution of 3-Nitropropionic acid derivatives in European Fabaceae, mainly within *Trib. Loteae DC*. and *Trib. Coronilleae*. In extracts of *Hippocrepis comosa*, 4-(3-nitropropanoyloxy) butanoic acid (**51**) (Figure 8) was identified, which may pose toxicity to livestock.

**Scheme 9:**
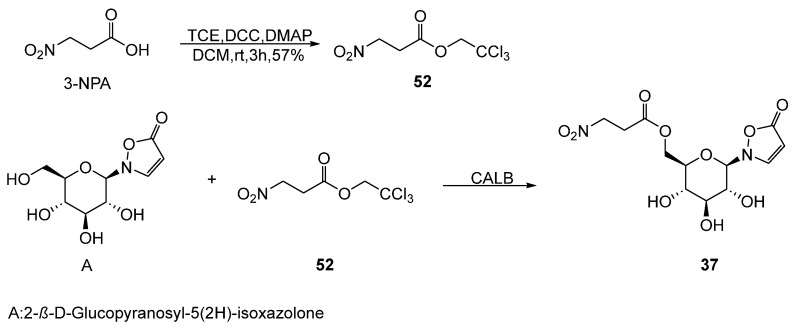
Biosynthetic pathway of compound **37**.

Becker et al. [67] elucidated the biosynthetic pathway from *β*-alanine to compound **37** and 3-Nitropropionic acid derivatives using stable isotope-labeled precursors. They identified 2,2,2-trichloroethyl-3-nitropropanoate (**52**) (Scheme 9) as a key intermediate in synthesizing compound **37** and confirmed the universality of this pathway across multiple Chrysomelidae beetle larvae species.

**Scheme 10:**
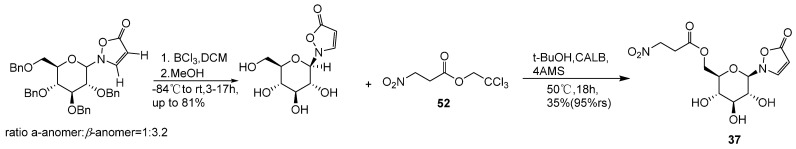
Chemical synthesis pathway of compound **37**.

Compound **52** can also be chemically converted to compound **37**. Tobias Becker et al. [68] developed a novel synthetic route utilizing cascade reactions to construct compound **37**. A critical step involved the enzymatic esterification of 3-Nitropropionic acid with 2-*β*-D-Glucopyranosyl-5(2H)-isoxazolone and 2-*α*-D-Glucopyranosyl-5(2H)-isoxazolone, yielding bioactive compound **37** (Scheme 10).

## 5. Other 3-Nitropropionic Acid Derivatives

**Figure 9:**
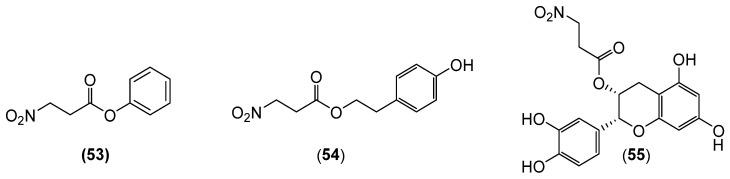
Structures of compounds **53**–**55**.

Phenyl 3-nitropropanoate (**53**) (Figure 9) is a 3-NPA derivative synthesized via the esterification of 3-NPA with phenol, resulting in a phenyl-substituted 3-nitropropanoate ester. The Bridgestone Corporation research group [69] employed compound **53** as a specialized coupling agent to synthesize poly(diene) and poly(diene) copolymers with reduced cold-flow properties.

**Scheme 11:**
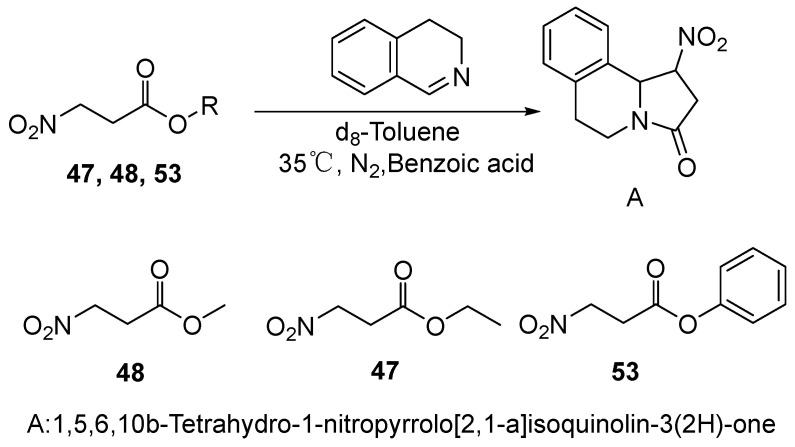
Rate-determining step involving compounds **47**, **48**, and **53**.

Pelletier et al. [70] (Scheme 11) employed cyclic imine as a model substrate to investigate the rate-determining step in the nitro-Mannich/lactamization reaction cascade by comparing the reaction kinetics of nitroesters with different ester groups (compounds **47**, **48**, and **53**) with 3,4-dihydroisoquinoline. Real-time monitoring via ^1^H NMR spectroscopy revealed that the reaction rate increased significantly with decreasing steric bulk of the ester moiety (phenyl > methyl > ethyl), clearly indicating that the lactamization step is rate-determining. These results support the hypothesis that the observed diastereocontrol originates from the favored pseudoequatorial orientation of the substituents during the lactamization process rather than during the reversible nitro-Mannich reaction. This study successfully developed a highly diastereoselective cascade reaction between methyl 2-alkyl-3-nitropropanoates and in situ generated imines, providing a novel synthetic approach to 1,3,5-trisubstituted-4-nitropyrrolidin-2-one derivatives.

Phomonitroester (**54**) (Figure 9) is derived from the endophytic fungi *Phomopsis* sp. PSU-D15 [2], marine fungi *Pestalotiopsis diploclisia* (BCC 35283) [71], and *Arthrinium* spp. [72]. Klaiklay et al. [73] isolated compound **54** from the mangrove-derived endophytic fungus *Phomopsis* sp. PSU-MA214. This compound exhibited selective cytotoxicity against human oral cancer cells (KB) with an IC_50_ value of 43 µg/mL. Ignjatović et al. [74] performed molecular docking simulations using Auto-dock v4.2, revealing compound **54**’s strong binding affinities to multiple receptors, particularly 3G7B (*Staphylococcus aureus gyrase* B) and 1F0K (*Escherichia coli* transferase).

Qiu et al. [75] isolated compound HQM-3 (**55**) (Figure 9) from *Indigofera stachyodes* (Fabaceae). Treatment with compound **55** resulted in 122.0% cell viability, surpassing the protective effects of the positive controls (e.g., bifendate and bicyclol tablets) against CCl_4_-induced hepatocyte damage in vitro. Hao et al. [76] developed methods to formulate compound **55** into injectables, lyophilized powders, oral liquids, tablets, and capsules, providing a scientific and technical foundation for novel hepatoprotective drugs. Compound **55**, a unique 3-nitropropanoate-linked flavonol derivative, derives its hepatoprotective activity from the 3-nitropropanoate substituent.

## 6. Conclusions

Since the first isolation of the 3-Nitropropionic acid derivative Hiptagin (**2**) in 1920 a total of 55 such compounds have been identified. Based on their structural characteristics, we have systematically classified these compounds into distinct categories. Among them, 3-Nitropropionic acid sugar esters, predominantly derived from leguminous plants, exhibit diverse bioactivities including antiviral, insecticidal, antibacterial, and antioxidant properties. However, among the 44 identified 3-Nitropropionic acid sugar esters, only approximately 25% have been pharmacologically characterized, with limited studies on their chemical syntheses, structural modifications, and mechanisms of action. 3-Nitropropionic acid non-sugar esters and other structural types show broad application potential in medicine, materials science, and agriculture. Linear derivatives, mostly small molecules, are widely used in chemical syntheses due to their reactivity and synthetic accessibility. However, studies on their pharmacological activities are sparse, and naturally occurring linear derivatives remain rare. Furthermore, significant gaps persist in the structural elucidation of linear and other 3-Nitropropionic acid derivatives, requiring further systematic exploration.

As a distinct class of nitro compounds, 3-Nitropropionic acid derivatives hold a unique position in medicinal chemistry. The nitro group, with its strong electron-withdrawing nature, significantly alters molecular electronic distribution, modulating reactivity and bioactivity. This electron-withdrawing effect can create localized electrophilic regions that facilitate interactions with biomacromolecules such as proteins and nucleic acids. Additionally, the selective toxicity of nitro compounds enables targeted action against specific pathogens or cancer cells [77]. Despite their remarkable pharmacological potential, nitro compounds are often associated with adverse effects and toxicity, such as carcinogenicity, hepatotoxicity, mutagenicity, and myelosuppression, which require careful consideration. During metabolic reduction in vivo, nitro groups may generate free radicals or reactive intermediates capable of reacting with DNA, proteins, and other biomolecules, leading to cellular damage or mutagenesis [78,79].

Similarly, while 3-NPA, as a privileged pharmacophore, demonstrates broad therapeutic potential in medicinal chemistry, its inherent toxicity requires cautious management. Through rational structural design and optimization, 3-Nitropropionic acid derivatives may be developed into efficacious therapeutic agents, particularly for oncology and antimicrobial/antiparasitic applications. Future research should prioritize balancing efficacy and toxicity to develop safer and more potent drugs, ultimately unlocking their full therapeutic potentials.

## Data Availability

No new data were created or analyzed in this study.

## Abbreviations

The following abbreviations are used in this manuscript:

3-NPA	3-Nitropropionic acid
HD	Huntington’s disease
ProNGF	Pro-nerve growth factor
Nrf2-ARE	Nuclear factor erythroid 2-related factor 2-Antioxidant Response Element
^13^C-NMR	Carbon-13 Nuclear Magnetic Resonance
MIC	Minimum inhibitory concentration
MBC	Minimum bactericidal concentration
HSV	Herpes simplex virus
BFCs	Bifunctional chelating agents
DAZA	1,4-Diazepane
GC-MS	Gas Chromatography-Mass Spectrometry
ICE	Interleukin-1β-Converting Enzyme
CED-3	Cell Death Abnormal-3
SARS-CoV-2	Severe Acute Respiratory Syndrome Coronavirus 2
FTY720	Fingolimod
PSU	Prince of Songkla University
BCC 35283	BIOTEC Culture Collection No. 35283
IC50	Half Maximal Inhibitory Concentration
DNA	Deoxyribonucleic Acid
THF	Tetrahydrofuran
TEA	Triethylamine
TEMPO	2,2,6,6-Tetramethylpiperidin-1-oxyl
TCE	Trichloroethylene
DCC	N,N′-Dicyclohexylcarbodiimide
DMAP	4-Dimethylaminopyridine
DCM	Dichloromethane
CALB	Candida antarctica Lipase B
BGC	Biosynthetic gene cluster
